# Mortality of Philadelphia, for January, February and March, 1854, Collated from the Record Kept at the Health Office

**Published:** 1854-05

**Authors:** Wilson Jewell


*Mortality of Philadelphia, for January, February and March, 1854, collated from the record kept at the Health Office.* By WILSON JEWELL, M. D.

The total of deaths for January, February and March of the present year, on record at the Health Office, amounts to 2580. This embraces a period of 91 days, or 13 weeks; and averages $28\frac{1}{3}$ deaths daily, or, to the population, estimating the same 415,000 as 1 to every 160.85. An increase of 4 per cent. over the deaths for the like period of 1853.

Exclusive of Still Born, External or Accidental causes, Old Age, Debility and Unknown, we have left 2140 deaths from recorded diseases, distinguished by 148 different names; many of them, it is true, almost as indefinite and as obscure for scientific purposes, as those set down under the head of debility or unknown.

The loose and imperfect plan of certification in cases of death is justly to be deplored; nor until there is a more discriminate system substituted for the present *generalizing* practice, can we have for reference an intelligible and profitable record. This vagueness of certification is not peculiar to Philadelphia; it prevails in other large cities, and has been elsewhere made the subject of remark. The City Inspector of New York, in his annual report to the Board of Aldermen, of the deaths in that city for 1852, complains of the want of precision on the part of physicians in assigning the causes of death, and suggests that the civil authorities should "early authorize some initiative step in the acquisition" of a measure which would enable him to furnish "yearly statements with an accuracy and fullness of detail, from which might be gathered a comprehensive and credible knowledge of all the occurrences of mortality presented for record."

Desirable as this change is, I am persuaded that no *dictum* of law can effect it. If ever we have a comprehensive and reliable registration of deaths, it must be done by the united sanction of the medical profession. Who are, or who ought to be interested in the perfecting of this branch of science, more than the members of the medical staff? They have both the ability and the means at hand to accomplish the desired end. But I am sorry to add, they lack the energy or the will to carry it out. The most stringent legal enactments on this head may be evaded, if they have not the approval of the profession. The best proof of this opinion is, that although we have on our statute book a "Registration act," it is neither obeyed nor honored. A dead letter on the legal record, it had far better be repealed, or else so modified that it may receive the approbation and enjoy the confidence of those whose duty it is and whose pride it should be to fulfil its laudable design.

The excess of deaths of males for the 1st quarter of 1854 is equal to 13 per cent. In the nervous diseases alone, it is equivalent to 35 per cent.

The excess of deaths among males prevails in most of the tables I have published since January, 1852. It is noticed also in the tables of other cities, thus giving it almost a fixed position in mortuary statistics.

Infantile mortality maintains its excess over more advanced life. The astounding number of deaths under one year, compared with the whole for the quarter, is worthy of a careful examination into the causes thereof. 1175, equal to 48 per cent. of the whole, exclusive of still born, died before the 5th year. While 579 of these, equal to 23½ per cent., were under one year of life. Diseases of the nervous system furnish 191. The organs of respiration 136. Sporadic diseases 121, and those of an epidemic form, 61.

1389 of all the deaths, or 52 per cent., were under 20 years of age. This calculation does not include the still born.

TABLE No. 1 exhibits a classified arrangement of the deaths for the quarter. The deaths in February exceeded those in January by 27 per cent., while in March they fell off 21 per cent.

The endemic diseases show quite a falling off from those of the two preceding years; equal to 42 per cent. for 1852, and 27 per cent. for 1853. In 1852 Small Pox was prevailing to a great extent, last year the deaths from Scarlet Fever for the same period, more than doubled those for the present year.

The diseases of the Organs of Respiration are set down at 754, an advance of 21 per cent. over those for the same months in 1853. But the comparison between those for 1852 and this year for the like period is so trifling as to be undeserving of notice.

It is curious to observe the uniformity which prevails from year to year, as to the total number of certain causes of death as registered for similar periods, for instance,

| | | | | | | | | | | |
|---|---|---|---|---|---|---|---|---|---|---|
| Unknown in 1852, | 1st quarter, | was | 65. | In | 1853, | 61, | and | in | 1854, | 62. |
| Digestive Organs " | " | " | 139. | " | " | 146, | " | " | " | 149. |
| Integumentary System | " | " | 4. | " | " | 6, | " | " | " | 4. |
| External Causes | " | " | 74. | " | " | 87, | " | " | " | 88. |
| Urinary Organs | " | " | 9. | " | " | 14, | " | " | " | 14. |

TABLE No. 2, CLASS 1st. The great falling off in Endemic and Epidemic diseases for this quarter over those for the same period of 1852 and 1853, especially in the exanthemata, is a subject for especial notice. Only 8 deaths from Small Pox, while in 1852 there were 258 ! Scarlet Fever furnishes 107, while last year, 1st quarter, there were 223. Croup 101, does not differ materially from last year. Measles has been very prevalent during January, February and March, but certainly very mild in its character, as only 21 deaths from it have been recorded.

Whooping Cough, which has prevailed to some considerable extent, gives 31 deaths. Quite an increase when compared with 7 in 1852 and 3 in 1853, 1st quarter of those years ; accurate certification, however, might show a different result. Pulmonary and Cerebral affections frequently follow Pertussis, terminating in death, and are likely to be charged to other causes.

CLASS 2d. Sporadic diseases. The only thing worthy of note here, is, that Debility and Marasmus tend to make up nearly 3-5ths of the deaths recorded under this head, from 26 distinct causes, amounting in all to 292, while Marasmus and Debility furnish 169 of them.

CLASS 3. Nervous Diseases. As usual, Convulsions occupy a prominent position in this class ; amounting to 178, or 33 per cent. of the whole number. Of these, 163 were under five years of age. The gradual increase of Convulsions from year to year is a subject that deserves the closest investigation. It possesses an interest extending far beyond the legitimate inquiry of the medical profession. The political economist, the philanthropist and the municipal authorities are all equally interested in solving the problem of the excess of infantile mortality in cities, of which Convulsions make up a very large proportion. A more enlarged cultivation of sanitary science, or the laws of health and life, by those who have the surveillance of our laws and ordinances, could not fail to be of incalculable advantage to the future well being of infant life.

Class 4th. Organs of Respiration. The mortality from this class of diseases, has increased 21 per cent. over that of a similar period for 1852. It is proper to observe, however, that last year there was a falling off of 20 per cent. below that for 1852.

Estimating the deaths from known diseases as recorded for the quarter, to be 2140, it will be found that this class constitutes 35 per cent. of the whole.

Consumption of the Lungs furnishes a high numeration in this table. It has credit for 385 deaths. Equal to 18 per cent. of the mortality from diseases, or 15 per cent. of the deaths from all causes. The excess of deaths from Consumption of the Lungs during this quarter was among males. Equivalent to 9 per cent.

Inflammation of the Bronchiæ and Lungs have produced 265 deaths; an increase of 32 per cent. over those for the 1st quarter of 1853. 30 per cent. of these deaths were in children within the 5th year. The severity of the past winter above that of 1852 and 1853 may be assigned as the cause for an increase of deaths from diseases of the respiratory organs.

Class 5th. There have been seventy-seven deaths from diseases of the Organs of Circulation; more than one-half, viz.: 42, are set down to "Disease of Heart." A proper term, but conveying very little information of a useful character, in the preparation of medical statistics. This generalizing is not peculiar to this class of diseases.

Table No. 3 gives the mortality at fifteen periods of life. By excluding the "Still Born," it appears that nearly one-half of all the deaths took place in infancy, or before the fifth year. That period of life between the ages of ten and fifteen seems to be the most healthy, contributing less than 1½ per cent. to the mortality for the quarter. The decennial period, between twenty and thirty, in all the tables I have published, save one, furnish the greatest outlet to human life among the adult population. 168, or 7 per cent. of the deaths, exclusive of "Still Born," were beyond 70 years of age. Of these, five were over one hundred years.

### TABLE NO. I.

*Deaths for the first quarter of* 1854 *classified.*

| | Jan. | Feb. | Mar. | Male. J. | Male. F. | Male. M. | Female. J. | Female. F. | Female. M. | Total. |
|---|---|---|---|---|---|---|---|---|---|---|
| 1 *Endemic & Contagious diseases* | | | | | | | | | | |
| Zymotic or Epidemic | 115 | 171 | 107 | 56 | 80 | 58 | 59 | 91 | 49 | 393 |
| 2 *Uncertain or general seat* | | | | | | | | | | |
| Sporadic diseases | 85 | 110 | 97 | 35 | 58 | 53 | 50 | 52 | 44 | 292 |
| 3 Nervous system | 125 | 205 | 186 | 83 | 122 | 108 | 42 | 83 | 78 | 516 |
| 4 Organs of Respiration | 250 | 280 | 224 | 128 | 146 | 121 | 122 | 134 | 103 | 754 |
| 5 " Circulation | 28 | 27 | 22 | 13 | 13 | 13 | 15 | 14 | 9 | 77 |
| 6 Digestive organs | 34 | 55 | 60 | 19 | 29 | 26 | 15 | 26 | 34 | 149 |
| 7 Urinary " | 1 | 6 | 7 | 1 | 6 | 7 | | | | 14 |
| 8 Organs of Generation | 9 | 7 | 9 | | | | 9 | 7 | 9 | 25 |
| 9 " Locomotion | 2 | 5 | 6 | 1 | 2 | 4 | 1 | 3 | 2 | 13 |
| 10 Integumentary system | 2 | | 2 | 1 | | 1 | 1 | | 1 | 4 |
| 11 Old age | 12 | 24 | 10 | 5 | 12 | 1 | 7 | 12 | 9 | 46 |
| 12 External causes | 21 | 41 | 26 | 7 | 26 | 16 | 14 | 15 | 10 | 88 |
| Still Born | 44 | 64 | 39 | 23 | 31 | 19 | 21 | 33 | 20 | 147 |
| Unknown | 21 | 29 | 12 | 15 | 22 | 8 | 6 | 7 | 4 | 62 |
| | 749 | 1024 | 807 | 387 | 547 | 435 | 362 | 477 | 372 | 2580 |

### TABLE NO. 2.

1. *Endemic and Contagious Diseases—Zymotic or Epidemic.*

| | Male. | Female. | Under 1 yr. | 1 to 2. | 2 to 5. | 5 to 10. | 10 to 15. | 15 to 20. | 20 to 30. | 30 to 40. | 40 to 50. | 50 to 60. | 60 to 70. | 70 to 80. | 80 to 90. | 90 to 100. | 100 to 110. | Total. |
|---|---|---|---|---|---|---|---|---|---|---|---|---|---|---|---|---|---|---|
| Cholera Infantum | 1 | | 1 | | | | | | | | | | | | | | | 1 |
| " morbus | | 1 | | | | | | | | | | 1 | | | | | | 1 |
| Croup | 53 | 48 | 12 | 23 | 56 | 10 | | | | | | | | | | | | 101 |
| Diarrhœa | 5 | 6 | 3 | 1 | | | | 1 | | 1 | 3 | 1 | | 1 | | | | 11 |
| Dysentery | 10 | 12 | 2 | 3 | 4 | 1 | | | 3 | 1 | 3 | 2 | 2 | 1 | | | | 22 |
| Erysipelas | 14 | 6 | 9 | 1 | 2 | | | 1 | 3 | 1 | | 1 | | 1 | 1 | | | 20 |
| Fever, Bilious | | 1 | | | | | | | | | | | | 1 | | | | 1 |
| " Continued | 1 | | | | | | | | | | | | | | 1 | | | 1 |
| " Intermittent | 1 | | | | | 1 | | | | | | | | | | | | 1 |
| " Nervous | | 2 | | | | | | 1 | | | 1 | | | | | | | 2 |
| " Puerperal | | 8 | | | | | | 2 | 1 | 4 | 1 | | | | | | | 8 |
| " Remittent | 1 | 3 | | | 3 | | | | | 1 | | | | | | | | 4 |
| " Scarlet | 50 | 57 | 14 | 20 | 49 | 22 | | 1 | | | 1 | | | | | | | 107 |
| " Typhoid | 21 | 14 | | 2 | 4 | 4 | 3 | 4 | 5 | 5 | 1 | 4 | 1 | 2 | | | | 35 |
| " Typhus | 10 | 7 | | | 1 | 2 | | 3 | 5 | 2 | 1 | 1 | 1 | 1 | | | | 17 |
| Measles | 12 | 9 | 5 | 6 | 7 | 3 | | | | | | | | | | | | 21 |
| Small Pox | 3 | 5 | 3 | 1 | | 1 | 1 | 1 | | 1 | | | | | | | | 8 |
| Varioloid | 1 | | | | | | | | 1 | | | | | | | | | 1 |
| Whooping Cough | 11 | 20 | 12 | 9 | 8 | 2 | | | | | | | | | | | | 31 |
| | 194 | 199 | 61 | 66 | 134 | 46 | 4 | 14 | 18 | 16 | 11 | 10 | 4 | 7 | 2 | | | 393 |

2. *Uncertain or General Seat—Sporadic Diseases.*

| | Male. | Female. | Under 1 yr. | 1 to 2. | 2 to 5. | 5 to 10. | 10 to 15. | 15 to 20. | 20 to 30. | 30 to 40. | 40 to 50. | 50 to 60. | 60 to 70. | 70 to 80. | 80 to 90. | 90 to 100. | 100 to 110. | Total. |
|---|---|---|---|---|---|---|---|---|---|---|---|---|---|---|---|---|---|---|
| Abscess | 5 | 1 | | | 1 | 1 | | | 1 | 2 | | 1 | | | | | | 6 |
| Cachexia | 1 | | | | | 1 | | | | | | | | | | | | 1 |
| Cancer | 1 | 4 | | | | | | | | 1 | 1 | 1 | 2 | | | | | 5 |
| " Breast | | 4 | | | | | | | | | 1 | 2 | 1 | | | | | 4 |
| Debility | 47 | 50 | 48 | 4 | 3 | 2 | 1 | 2 | 1 | 7 | 4 | 6 | 7 | 9 | 3 | | | 97 |
| Disease, Complicated | | 1 | | | | | | | | | | | 1 | | | | | 1 |
| " Breast | | 1 | | | | | | | | | | | | 1 | | | | 1 |
| Dropsy | 14 | 17 | 1 | 3 | 4 | | | 3 | 4 | 5 | 2 | 1 | 1 | 3 | 3 | 1 | | 31 |
| Enlargem't Parotid Gland | 1 | | | | | | | | | | | | 1 | | | | | 1 |
| Fungus Hematodes | | 1 | | | | | | | | 1 | | | | | | | | 1 |
| Gangrene | 1 | 3 | 2 | | | | | | 1 | | | | | 1 | | | | 4 |
| Gout | 1 | | | | | | | | | | | 1 | | | | | | 1 |
| Hemorrhage | 3 | | 1 | | 1 | | | | | | 1 | | | | | | | 3 |
| " Mouth | | 1 | 1 | | | | | | | | | | | | | | | 1 |
| Inanition | 10 | 7 | 12 | 1 | | | | 1 | | | | | 1 | | 2 | | | 17 |
| Inflammation | 2 | | | | | | | | | | | 2 | | | | | | 2 |
| Malformation | 3 | 2 | 5 | | | | | | | | | | | | | | | 5 |
| Marasmus | 40 | 32 | 38 | 11 | 11 | 2 | | | | | 4 | 3 | 2 | | 1 | | | 72 |
| Mortification | | 1 | | | | | | 1 | | | | | | | | | | 1 |
| Schirrhus | | 1 | | | | | | | | | | | 1 | | | | | 1 |
| Scrofula | 12 | 10 | 6 | 4 | 5 | 2 | | | 1 | 3 | | | 1 | | | | | 22 |
| Tabes Mesenterica | 4 | 4 | 3 | 1 | 3 | 1 | | | | | | | | | | | | 8 |
| Tumour | | 1 | 1 | | | | | | | | | | | | | | | 1 |
| " Abdominal | | 2 | | | | | | | | | | | 2 | | | | | 2 |
| Ulceration of Neck | | 1 | 1 | | | | | | | | | | | | | | | 1 |
| " Throat | 1 | 2 | 2 | | | 1 | | | | | | | | | | | | 3 |
| | 146 | 146 | 121 | 24 | 28 | 10 | 1 | 7 | 8 | 19 | 13 | 17 | 20 | 14 | 9 | 1 | | 292 |

3. *Nervous Diseases.*

| | Male. | Female. | Under 1 yr. | 1 to 2. | 2 to 5. | 5 to 10. | 10 to 15. | 15 to 20. | 20 to 30. | 30 to 40. | 40 to 50. | 50 to 60. | 60 to 70. | 70 to 80. | 80 to 90. | 90 to 100. | 100 to 110. | Total. |
|---|---|---|---|---|---|---|---|---|---|---|---|---|---|---|---|---|---|---|
| Abscess of Brain | 1 | | 1 | | | | | | | | | | | | | | | 1 |
| Apoplexy | 13 | 10 | | | | | | | 2 | | 3 | 9 | 5 | 3 | 1 | | | 23 |
| Concussion of Brain | 2 | 1 | | 1 | 1 | | | | | | 1 | | | | | | | 3 |
| Congestion " | 28 | 18 | 13 | 5 | 8 | 7 | 2 | 1 | 1 | 1 | 2 | 2 | 3 | | 1 | | | 46 |
| Compression " | | 1 | 1 | | | | | | | | | | | | | | | 1 |
| Convulsions | 117 | 61 | 109 | 30 | 24 | 4 | 2 | | 3 | 2 | 3 | | | 1 | | | | 178 |
| Disease of Brain | 16 | 13 | 6 | 5 | 8 | 1 | | 1 | | 3 | 1 | 1 | 1 | 2 | | | | 29 |
| Dropsy " | 42 | 34 | 31 | 19 | 22 | 3 | | | | | | 1 | | | | | | 76 |
| Effusion " | 12 | 12 | 8 | 7 | 4 | 1 | | 1 | 2 | | | 1 | | | | | | 24 |
| Epilepsy | 4 | 2 | | | | | | | 1 | 1 | 1 | 2 | 1 | | | | | 6 |
| Hysteria | 1 | | | 1 | | | | | | | | | | | | | | 1 |
| Inflammation of Brain | 56 | 34 | 22 | 15 | 16 | 11 | 2 | 5 | 4 | 2 | 6 | 2 | 3 | 2 | | | | 90 |
| Mania | | 1 | | | | | | | | 1 | | | | | | | | 1 |
| " a Potu | 5 | 2 | | | | | | | 1 | 2 | 1 | 1 | 2 | | | | | 7 |
| Palsy | 7 | 13 | | | | | | | 1 | 2 | 3 | | 3 | 8 | 3 | | | 20 |
| Softening of Brain | 5 | 1 | | | 1 | 1 | | | 1 | | 3 | | | | | | | 6 |
| Tetanus | 4 | | | | | 1 | | 1 | | 1 | | 1 | | | | | | 4 |
| | 313 | 203 | 191 | 83 | 84 | 29 | 6 | 9 | 16 | 15 | 24 | 20 | 18 | 16 | 5 | | | 516 |

### 4. *Organs of Respiration.*

| | Male. | Female. | Under 1 yr. | 1 to 2. | 2 to 5. | 5 to 10. | 10 to 15. | 15 to 20. | 20 to 30. | 30 to 40. | 40 to 50. | 50 to 60. | 60 to 70. | 70 to 80. | 80 to 90. | 90 to 100. | Total. |
|---|---|---|---|---|---|---|---|---|---|---|---|---|---|---|---|---|---|
| Abscess of Lungs | 3 | 3 | 2 | | 1 | | | | | | | 3 | | | | | 6 |
| Asthma | 4 | 4 | 1 | | | | | | | 1 | 2 | | 2 | 2 | | | 8 |
| Congestion of Lungs | 12 | 12 | 13 | 3 | 3 | | 1 | | 2 | | | 1 | 1 | | | | 24 |
| Consumption " | 202 | 183 | 8 | 6 | 7 | 7 | 5 | 20 | 125 | 86 | 49 | 37 | 24 | 8 | 3 | | 385 |
| Coryza | | 1 | 1 | | | | | | | | | | | | | | 1 |
| Disease of Breast | | 1 | 1 | | | | | | | | | | | | | | 1 |
| " Chest | 1 | 1 | 1 | | | | | | | | 1 | | | | | | 2 |
| " Lungs | 4 | 6 | 1 | 3 | | | | | 3 | 1 | 2 | | | | | | 10 |
| Dropsy of Chest | 5 | 4 | | | | 1 | 1 | | 3 | | 1 | | 1 | 2 | | | 9 |
| " Pleura | | 1 | | | | | | | | | | 1 | | | | | 1 |
| Edema of Lungs | | 1 | | | | | | | | 1 | | | | | | | 1 |
| Effusion of Chest | 1 | 4 | 1 | | 2 | 1 | | | 1 | | | | | | | | 5 |
| " Lungs | | 1 | | 1 | | | | | | | | | | | | | 1 |
| Empyema | | 1 | | | | | | | | 1 | | | | | | | 1 |
| Emphysema | | 1 | 1 | | | | | | | | | | | | | | 1 |
| Enlargem't Thymic Gland | 1 | | 1 | | | | | | | | | | | | | | 1 |
| Inflamm'n of Bronchiæ | 57 | 48 | 57 | 21 | 11 | 1 | | 1 | | 1 | 4 | | 2 | 6 | 1 | | 105 |
| " Chest | 2 | | | | 1 | | | | 1 | | | | | | | | 2 |
| " Fauces | | 1 | | | 1 | | | | | | | | | | | | 1 |
| " Larynx | 5 | 3 | 3 | 1 | 1 | 3 | | | | | | | | | | | 8 |
| " Lungs | 85 | 75 | 44 | 36 | 22 | 5 | | 1 | 9 | 7 | 2 | 13 | 7 | 9 | 5 | | 160 |
| " Pleura | 5 | 2 | | | | | | | 1 | 2 | 1 | 1 | 1 | 1 | | | 7 |
| " Throat | 2 | 2 | 1 | 1 | 1 | 1 | | | | | | | | | | | 4 |
| Hemorrhage of Lungs | 6 | 3 | | | | | | | 2 | 3 | 1 | 3 | | | | | 9 |
| Tuberculosis | | 1 | | | | | | | | 1 | | | | | | | 1 |
| | 395 | 359 | 136 | 72 | 50 | 19 | 7 | 22 | 147 | 104 | 63 | 59 | 38 | 28 | 9 | | 754 |

### 5. *Organs of Circulation.*

| | Male. | Female. | Under 1 yr. | 1 to 2. | 2 to 5. | 5 to 10. | 10 to 15. | 15 to 20. | 20 to 30. | 30 to 40. | 40 to 50. | 50 to 60. | 60 to 70. | 70 to 80. | 80 to 90. | 90 to 100. | 100 to 110. | Total. |
|---|---|---|---|---|---|---|---|---|---|---|---|---|---|---|---|---|---|---|
| Anæmia | 1 | 1 | 1 | | | | | | | | | 1 | | | | | | 2 |
| Angina Pectoris | 1 | | | | | | | | | | | | 1 | | | | | 1 |
| Cyanosis | 5 | 4 | 9 | | | | | | | | | | | | | | | 9 |
| Disease of Heart | 22 | 20 | 5 | 1 | 3 | 3 | 1 | 1 | 2 | 2 | 6 | 5 | 7 | 6 | | | | 42 |
| Dropsy " | | 4 | | | | | | | 1 | | 1 | 1 | | 1 | | | | 4 |
| Effusion " | 1 | 1 | | | | | | | 1 | | 1 | | | | | | | 2 |
| Enlargem't " | 3 | 6 | | | | | 2 | 1 | 1 | 2 | 2 | | 1 | | | | | 9 |
| Inflammat'n " | | 1 | | | | | | | | | | 1 | | | | | | 1 |
| " Veins | 1 | | | | | | | | | | 1 | | | | | | | 1 |
| Malformation of Heart | 3 | 1 | 4 | | | | | | | | | | | | | | | 4 |
| Ossification " | 1 | | | | | | | | | | | | | 1 | | | | 1 |
| Rheumatism " | 1 | | | | 1 | | | | | | | | | | | | | 1 |
| | 39 | 38 | 19 | 1 | 4 | 3 | 3 | 2 | 5 | 4 | 11 | 8 | 9 | 8 | | | | 77 |

6. *Digestive Organs.*

| | Male. | Female. | Under 1 yr. | 1 to 2. | 2 to 5. | 5 to 10. | 10 to 15. | 15 to 20. | 20 to 30. | 30 to 40. | 40 to 50. | 50 to 60. | 60 to 70. | 70 to 80. | 80 to 90. | 90 to 100. | 100 to 110. | Total. |
|---|---|---|---|---|---|---|---|---|---|---|---|---|---|---|---|---|---|---|
| Abscess of Intestines | 1 | | | | | | | | | | | | 1 | | | | | 1 |
| " Liver | 2 | 1 | | | | | | | 1 | | | 1 | | 1 | | | | 3 |
| Anorexia | | 1 | 1 | | | | | | | | | | | | | | | 1 |
| Cancer of Liver | 1 | | | | | | | | | | 1 | | | | | | | 1 |
| " Stomach | 3 | 3 | | | | | | | | | 2 | 2 | 2 | | | | | 6 |
| " Tongue | 1 | | | | | | | | | | | 1 | | | | | | 1 |
| Cancrum Oris | | 1 | | | 1 | | | | | | | | | | | | | 1 |
| Cirrhosis of Liver | 4 | | | | | | | | 1 | | 2 | 1 | | | | | | 4 |
| Congestion " | 1 | 1 | | | | | 1 | | | | | | 1 | | | | | 2 |
| Colica Pictonum | 1 | | | | | | | | | | 1 | | | | | | | 1 |
| Consumption of Bowels | 2 | | | | | | | | | 1 | | | | | 1 | | | 2 |
| Disease of Bowels | 1 | | 1 | | | | | | | | | | | | | | | 1 |
| " Stomach & do. | 1 | | | | | | | | | 1 | | | | | | | | 1 |
| " Liver | 4 | 6 | 2 | | | | | | 2 | 1 | 1 | 1 | 1 | 1 | 1 | | | 10 |
| Dropsy Abdominal | 3 | 6 | | | | | | | | 2 | 4 | 1 | 2 | | | | | 9 |
| Hemorrhage from Stomach | | 1 | 1 | | | | | | | | | | | | | | | 1 |
| Hernia Strangulated | | 4 | | | | | | | | | 2 | 1 | | 1 | | | | 4 |
| " Umbilical | 1 | | | | | | | | | | | 1 | | | | | | 1 |
| Ileus | 1 | | | | | | | | | | 1 | | | | | | | 1 |
| Inflammation of Liver | 3 | 7 | | 1 | | | | | 2 | 2 | 1 | | 2 | 2 | | | | 10 |
| " Peritoneum | 3 | 13 | 1 | | | | | 2 | 2 | 4 | 4 | 1 | 1 | | 1 | | | 16 |
| " Stomach & Bowels | 27 | 23 | 6 | 7 | 8 | 3 | 3 | 3 | 4 | 2 | 3 | 3 | 5 | 2 | 1 | | | 50 |
| Jaundice | 5 | 3 | 2 | | 2 | | | | | | 1 | 2 | | 1 | | | | 8 |
| Obstruction of Bowels | 1 | | 1 | | | | | | | | | | | | | | | 1 |
| Perforation " | | 1 | | | | | | | 1 | | | | | | | | | 1 |
| Schirrus Stomach | 2 | 2 | | | | | | | | | 1 | | 2 | 1 | | | | 4 |
| Teething | 2 | 1 | 2 | 1 | | | | | | | | | | | | | | 3 |
| Ulceration of Bowels | 3 | 1 | 2 | | | | | | | 1 | | | | | 1 | | | 4 |
| " Liver | 1 | | | | | | | | | 1 | | | | | | | | 1 |
| | 74 | 75 | 19 | 9 | 11 | 3 | 4 | 5 | 13 | 16 | 23 | 15 | 17 | 9 | 5 | | | 149 |

7. *Urinary Organs.*

| | Male. | Female. | Under 1 yr. | 1 to 2. | 2 to 5. | 5 to 10. | 10 to 15. | 15 to 20. | 20 to 30. | 30 to 40. | 40 to 50. | 50 to 60. | 60 to 70. | 70 to 80. | 80 to 90. | 90 to 100. | 100 to 110. | Total. |
|---|---|---|---|---|---|---|---|---|---|---|---|---|---|---|---|---|---|---|
| Albuminuria | 2 | | | | | | | | 1 | 1 | | | | | | | | 2 |
| Diabetes | 2 | | | | | | 1 | 1 | | | | | | | | | | 2 |
| Disease of Kidneys, | 3 | | | 1 | | 1 | | | | | | | 1 | | | | | 3 |
| Inflammation of do. | 3 | | | | | | | | 1 | | 1 | 1 | | | | | | 3 |
| " of Bladder, | 3 | | | | | | | | | 1 | 1 | | 1 | | | | | 3 |
| Stone in " | 1 | | | | | | 1 | | | | | | | | | | | 1 |
| | 14 | | | 1 | | 1 | 2 | 1 | 2 | 2 | 2 | 1 | 2 | | | | | 14 |

### 8. *Organs of Generation.*

| | Male. | Female. | Under 1 yr. | 1 to 2. | 2 to 5. | 5 to 10. | 10 to 15. | 15 to 20. | 20 to 30. | 30 to 40. | 40 to 50. | 50 to 60. | 60 to 70. | 70 to 80. | 80 to 90. | 90 to 100. | 100 to 110. | Total. |
|---|---|---|---|---|---|---|---|---|---|---|---|---|---|---|---|---|---|---|
| Cancer of Uterus, | | 7 | | | | | | | | 1 | 3 | 1 | 1 | | 1 | | | 7 |
| Child Bed, | | 1 | | | | | | | | 1 | | | | | | | | 1 |
| Dropsy Ovarian, | | 2 | | | | | | | 1 | | 1 | | | | | | | 2 |
| Hemorrhage of Uterus, | | 7 | | | | | | | 2 | 2 | 2 | | 1 | | | | | 7 |
| Inflammation of " | | 3 | | | | | | | 2 | 1 | | | | | | | | 3 |
| Puerperal Convulsions, | | 4 | | | | | | | 4 | | | | | | | | | 4 |
| Tumour Ovarian, | | 1 | | | | | | | | | | | 1 | | | | | 1 |
| | | 25 | | | | | | | 9 | 5 | 6 | 1 | 3 | | 1 | | | 25 |

### 9. *Organs of Locomotion.*

| | Male. | Female. | Under 1 yr. | 1 to 2. | 2 to 5. | 5 to 10. | 10 to 15. | 15 to 20. | 20 to 30. | 30 to 40. | 40 to 50. | 50 to 60. | 60 to 70. | 70 to 80. | 80 to 90. | 90 to 100. | 100 to 110. | Total. |
|---|---|---|---|---|---|---|---|---|---|---|---|---|---|---|---|---|---|---|
| Gangrene of Foot | | 1 | | | | | | | | | | | | 1 | | | | 1 |
| Injury of Spine | 1 | | | | | | | | | | | 1 | | | | | | 1 |
| Necrosis | | 1 | | | | | | | | | | | 1 | | | | | 1 |
| Rheumatism | 4 | 3 | | | | 1 | 1 | 1 | 1 | | | | 2 | 1 | | | | 7 |
| Spina Bifida | 2 | 1 | 2 | | 1 | | | | | | | | | | | | | 3 |
| | 7 | 6 | 2 | | 1 | 1 | 1 | 1 | 1 | | | 1 | 3 | 2 | | | | 13 |

### 10. *Integumentary System.*

| | Male. | Female. | Under 1 yr. | 1 to 2. | 2 to 5. | 5 to 10. | 10 to 15. | 15 to 20. | 20 to 30. | 30 to 40. | 40 to 50. | 50 to 60. | 60 to 70. | 70 to 80. | 80 to 90. | 90 to 100. | 100 to 110. | Total. |
|---|---|---|---|---|---|---|---|---|---|---|---|---|---|---|---|---|---|---|
| Disease of Skin | 1 | | 1 | | | | | | | | | | | | | | | 1 |
| Purpura Hemorrhagica | 1 | 1 | 1 | | 1 | | | | | | | | | | | | | 2 |
| Scurvy | | 1 | | 1 | | | | | | | | | | | | | | 1 |
| | 2 | 2 | 2 | 1 | 1 | | | | | | | | | | | | | 4 |

### 11. *Old Age.*

| | Male. | Female. | Under 1 yr. | 1 to 2. | 2 to 5. | 5 to 10. | 10 to 15. | 15 to 20. | 20 to 30. | 30 to 40. | 40 to 50. | 50 to 60. | 60 to 70. | 70 to 80. | 80 to 90. | 90 to 100. | 100 to 110. | Total. |
|---|---|---|---|---|---|---|---|---|---|---|---|---|---|---|---|---|---|---|
| Old Age | 18 | 28 | | | | | | | | | | | 4 | 11 | 17 | 9 | 5 | 46 |

12. *External Causes.*

| | Male. | Female. | Under 1 yr. | 1 to 2. | 2 to 5. | 5 to 10. | 10 to 15. | 15 to 20. | 20 to 30. | 30 to 40. | 40 to 50. | 50 to 60. | 60 to 70. | 70 to 80. | 80 to 90. | 90 to 100. | 100 to 110. | Total. |
|---|---|---|---|---|---|---|---|---|---|---|---|---|---|---|---|---|---|---|
| Asphyxia | 6 | 4 | 9 | 1 | | | | | | | | | | | | | | 10 |
| Burns | 5 | 17 | | 3 | 12 | 1 | 1 | 2 | | 1 | | | | 1 | 1 | | | 22 |
| Casualties | 21 | 7 | 1 | | 2 | 1 | 1 | 1 | 3 | 10 | 3 | 3 | 1 | 2 | | | | 28 |
| Drowned | 7 | | | | | | | | | | 4 | 2 | 1 | | | | | 7 |
| Fracture of Skull | 1 | | | | | 1 | | | | | | | | | | | | 1 |
| Gunshot Wound | | 1 | | | | | 1 | | | | | | | | | | | 1 |
| Intemperance | 2 | 5 | | | | | | | 1 | 5 | 1 | | | | | | | 7 |
| Poison | | 1 | | | | | | | 1 | | | | | | | | | 1 |
| Suffocation | 5 | 2 | 3 | | 1 | | 1 | | | | | | 2 | | | | | 7 |
| Suicide | 2 | 2 | | | | | | 1 | 1 | | 2 | | | | | | | 4 |
| | 49 | 39 | 13 | 4 | 15 | 3 | 4 | 4 | 6 | 16 | 10 | 5 | 4 | 3 | 1 | | | 88 |
| Still Born | 73 | 74 | 147 | | | | | | | | | | | | | | | 147 |
| Unknown | 45 | 17 | 15 | 4 | 3 | 1 | 1 | | 8 | 6 | 8 | 4 | 6 | 4 | 1 | 1 | | 62 |

### TABLE NO. 3.

*Deaths for the First Quarter of 1854, at fifteen distinct periods of life.*

| | |
|---|---|
| Under 1 year, | 579 |
| 1 to 2 | 265 |
| 2 to 5 | 331 |
| 5 to 10 | 116 |
| 10 to 15 | 33 |
| 15 to 20 | 65 |
| 20 to 30 | 233 |
| 30 to 40 | 203 |
| 40 to 50 | 171 |
| 50 to 60 | 141 |
| 60 to 70 | 128 |
| 70 to 80 | 102 |
| 80 to 90 | 50 |
| 90 to 100 | 11 |
| 100 to 110 | 5 |
| | 2433 |
| Still Born | 147 |
| Total, | 2580 |

Included within the foregoing tables, were 159 from the Blockley Alms House; also, 163 Blacks, and 20 from the country, as follows:

| | January. | February. | March. | Total. |
|---|---|---|---|---|
| Almshouse, | 46 | 70 | 43 | 159 |
| Blacks, | 58 | 50 | 55 | 163 |
| Country, | 7 | 9 | 4 | 20 |
| Total, | | | | 342 |

